# Questionnaire for the assessment of adherence barriers of intravitreal therapy: the ABQ-IVT

**DOI:** 10.1186/s40942-021-00311-x

**Published:** 2021-06-02

**Authors:** Sabrina Müller, Sophia Junker, Thomas Wilke, Albrecht Lommatzsch, Alexander K. Schuster, Hakan Kaymak, Christoph Ehlken, Focke Ziemssen

**Affiliations:** 1 IPAM e.v, University of Wismar, University of Applied Sciences, Alter Holzhafen 19, 23966 Wismar, Germany; 2 Ingress-Health HWM GmbH, Alter Holzhafen 19, 23966 Wismar, Germany; 3 Augenzentrum Am St. Franziskus-Hospital Münster, Hohenzollerning 74, 48145 Münster, Germany; 4 MORE Reading Center, Augenklinik Und Poliklinik Universitätsmedizin Mainz, Langenbeckstr. 1, 55131 Mainz, Germany; 5 Augenchirugie Clinic, Theo-Champion-Str. 1, 40549 Düsseldorf, Germany; 6 Universitaetsklinikum Schleswig-Holstein Kiel, Arnold-Heller-Straße 3, 24105 Kiel, Germany; 7 Universitäts-Augenklinik Tübingen, Elfriede-Aulhorn-Straße 7, 72076 Tübingen, Germany

**Keywords:** Adherence, Non-adherence, Adherence barriers, Age-related macular degeneration (AMD), Diabetic macular edema (DME), Intravitreal anti-VEGF injection (IVT), Adherence Barriers Questionnaire (ABQ), Patient questionnaire

## Abstract

**Objective:**

To develop and validate a questionnaire for the investigation of non-adherence (NA) barriers in patients receiving intravitreal injection (IVT).

**Design:**

Questionnaire development and cross-sectional patient survey combined with a retrospective medical chart review.

**Participants:**

German patients with neovascular age-related macular degeneration (nAMD) or diabetic macular edema (DME) receiving anti-vascular endothelial growth factor (anti-VEGF) treatment via IVT.

**Methods:**

The previously validated (indications: atrial fibrillation, human immunodeficiency virus, chronic inflammatory lung disease) Adherence Barriers Questionnaire (ABQ) was revised according to specifications of IVT, within the framework of an expert panel. The ABQ-IVT, which initially consisted of 24 items formulated as statements (4-point-Likert-scale ranging from “strongly agree” to “strongly disagree”), was applied in a cross-sectional survey. Evaluation of the questionnaire included an assessment of internal consistency and factor analysis. The occurrence of potential barriers in the patient sample was evaluated using descriptive statistics. To identify patient subpopulations, hierarchical cluster analysis was performed using ABQ-IVT answers as predictors. Due to difficulties in capturing NA as an external criterion, the evaluation of the questionnaire was limited to its internal validity and reliability.

**Main outcome measures:**

Patients’ answers to the ABQ-IVT questionnaire and interviews.

**Results:**

Of 253 patients, 234 (92%) were able to complete the ABQ-IVT questionnaire. Within the reliability analysis, the ABQ-IVT was reduced to 17 items. The condensed questionnaire demonstrated good internal consistency (Cronbach’s alpha = 0.78), and factor analysis showed no evidence for subscales of the questionnaire. Nearly half of the patients (49%) reported being affected by at least three different barriers. On average, a patient was affected by 3.1 barriers. The most frequently reported barriers were “Challenge due to time commitment of physician visits” (45% of the patients), “Depression” (29%) and “Travel and opportunity costs” (27%). Cluster analysis identified six patient subpopulations, each affected by different sets of barriers and differed regarding their patient characteristics.

**Conclusions:**

The ABQ-IVT is a practical and reliable instrument for identifying patient-specific barriers to IVT treatment adherence. In practice, the questionnaire may be useful in assessing whether individual patients are at higher risk of NA due to specific adherence barriers. Aside from better awareness, this allows earlier interventions, though these still need to be validated. Patient subpopulations face different barriers and may, therefore, need distinct preventative care.

**Supplementary Information:**

The online version contains supplementary material available at 10.1186/s40942-021-00311-x.

## Background

Anti-vascular endothelial growth factor (anti-VEGF) therapy currently represents the treatment standard for neovascular age-related macular degeneration (nAMD) and diabetic macular edema (DME) [1–3]. Many randomized controlled clinical trials have shown the beneficial effect of intravitreal anti-VEGF therapy (IVT) regarding the improvement and maintenance of visual acuity (VA) in patients affected by DME or nAMD [4–8]. However, real-world studies generally fail to reproduce these results, reporting stabilization or slight improvement of VA maintained only for shorter periods of time [9–11]. In these studies, more frequent physician visits and intravitreal injections were associated with greater effectiveness of IVT. One of the major factors influencing the number of administered injections is patients' adherence to treatment and monitoring.

Adherence to treatment is defined as the extent to which a patient's medicine-taking behavior corresponds with recommendations communicated by a health care provider. In view of the invasive character of intravitreal therapy, there is the peculiarity that the extent of non-adherence can be exactly determined, as it is usually known to the ophthalmologists how often the active substance was in fact administered. Adequate adherence is essential for realizing the potential benefits of medication-based treatments [12–14]. Nonetheless, 25–50% of the general patient population do not adhere to recommended treatment regimens [15–17]. In particular, patients with chronic diseases who are dependent on long-term therapy experience increased adherence difficulties [12]. Therapy adherence can be distinguished from non-persistence, where the treatment or monitoring is stopped or omitted for a longer period of time. The field of non-persistence seems to be a subject that is still completely underexposed. It is a demanding problem to assess patients and their difficulties which do not return to the follow-up in the routine and are therefore invisible to the treating physician. Non-persistent patients may be more reluctant to take part in scientific surveys, patients with cognitive issues may not be amenable, and deceased persons can no longer be interviewed. Nevertheless, the knowledge of the poor outcomes in the spontaneous course of those diseases gives an idea of how important it is to avoid the termination of a necessary therapy as the maximum form of undertreatment [18]. Inadequate adherence also negatively affects long-term outcomes in nAMD patients [19–21]. An observational study on adherence with DME and nAMD treatment showed that up to one-third of patients starting IVT either discontinue therapy or do not adhere to the recommended treatment regimen [22]. As adherence largely affects outcomes, and current methods for improving adherence of patients with chronic health problems are not effective enough to realize the full potential benefit of the respective therapies, ways to improve patients' adherence are urgently needed [17]. Barriers to medication adherence are complex and therefore require multifactorial solution approaches [16, 18, 23]. One approach is to identify the underlying reasons for non-adherence (NA) in patients treated with IVT. Until now, there is no reliable measure that can detect barriers to IVT adherence in everyday clinical practice.

This study sought to construct a questionnaire that can easily be used to differentially assess adherence barriers in patients receiving IVT. The "Adherence Barriers Questionnaire" (ABQ) is an instrument originally developed by Müller et al*.* to elicit barriers to treatment adherence in atrial fibrillation [24, 25] and has been adapted to assess adherence in several other indications such as human immunodeficiency virus and chronic obstructive pulmonary disease [26, 27]. The ABQ and its adaptations have been shown to be practical, reliable, and valid instruments to elicit barriers to treatment adherence in chronic indications with self-administered medication. The ABQ covers difficulties usually experienced by patients with chronic conditions and can be applied across indications, as it assesses universal barriers to treatment adherence such as lack of trust in the treating physician, fear of side effects, and depressive moods. However, different indications implicate distinct adherence hurdles, depending on the characteristics of the applied therapy. Our aim was to adapt the ABQ to the specific needs of nAMD/DME patients receiving IVT and to validate the questionnaire in a clinical setting.

## Methods

### Content validity/development of questionnaire

The first version of the questionnaire was adapted from a more general questionnaire, developed by Müller et al. in 2015, which has already been validated and established to record and assess the adherence for other diseases and routes of application [25]. The response structure was kept with each item being formulated as a statement. Answers were recorded on a 4-point Likert scale, on which patients had the option to choose between the following response options: 1—"strongly agree", 2—"generally agree", 3—"generally disagree" and 4—"strongly disagree". Items stating the presence of a certain barrier were reverse-coded, so that a higher score indicated a higher influence of a certain adherence barrier in the patient's perception.

An initial literature review was conducted within the scope of the adaptation process. No suitable patient-reported outcomes (PROs) addressing barriers of adherence had previously been published for IVT. Suitability of existing ABQ items and relevance of additional components was discussed within an expert panel consisting of clinicians with extensive experience regarding the treatment of nAMD/DME. Special focus was put on the relevance of items with respect to patients' adherence, clarity of phrasing, potential redundancy, and completeness of selected items. The preliminary ABQ-IVT was qualitatively assessed in comprehensive interviews with 15 focus patients. Patients were asked to rate the importance/relevance of each item and the completeness of the list, describing additional items to be included in the questionnaire if they felt that important factors were missing. The qualitative assessment additionally evaluated the appropriateness, redundancy/distinctiveness, and unbiasedness of questions as well as clarity of wording. Finally, respondent burden of the preliminary ABQ-IVT was measured by evaluating the time needed for completion of the questionnaire and identifying questions which patients were unwilling to answer. Only relevant, easily understandable, non-redundant items were included in the ABQ-IVT draft.

### Patient characteristics and psychometric properties of the ABQ-IVT (Questionnaire validation)

Patient characteristics, including sociographic information, information related to the nAMD/DME condition or the IVT treatment, and comorbidities were descriptively analyzed. Frequency analysis was applied for all categorical variables, reporting the number and percentage of patients for each category. For continuous variables, summary statistics, including mean and standard deviation (SD), were reported.

The utility of the ABQ-IVT was evaluated as follows: Firstly, the properties of each separate item were assessed. The clarity and appropriateness of questions can be estimated by examining missing data, as items with a disproportionally high share of missing data indicate overburdening of the respondent regarding sensibility or understanding. Consideration of floor and ceiling effects can provide information on the redundancy of items. Exclusion of items with a high endorsement rate for one answer option was discussed as these add little value to the index. Secondly, internal reliability was evaluated by calculating Cronbach's α, with 0.7 being the minimum accepted value for exploratory research [28]. The exclusion of an item was considered if its removal from the questionnaire led to considerable improvement of the Cronbach's α value. Split-half reliability was assessed based on the Spearman-Brown prediction formula. A Spearman-Brown coefficient of ≥ 0.7 was considered satisfactory. Furthermore, the item-total correlation was examined, with an accepted correlation value of 0.2–0.8 [29]. At the time of the study start, no tool assessing the degree of adherence to IVT in DME or nAMD patients was available, which could have been used to assess the converging validity of the ABQ-IVT. To examine external validity nonetheless, the correlation of ABQ scores with self-reported treatment adherence and persistence was examined using Mann–Whitney-U-tests. After a final decision regarding the exclusion of certain items had been made based on the evaluation of item properties and reliability, a factor analysis with varimax rotation was applied for the identification of potential subscales of the ABQ-IVT.

### Patient inclusion

Psychometric properties of the initially developed version were evaluated within a cross-sectional multi-center study of patients with nAMD or DME in Germany. General patient inclusion criteria were a confirmed diagnosis with either nAMD or DME and the start of IVT between 0.5 and 3 years prior to study inclusion. Based on a nation-wide list of more than 500 ophthalmologists, potential study sites treating patients with IVT across Germany were invited to take part in the study. Participating study investigators (SIs) were asked to generate a list with all patients who met the general inclusion criteria and for whom basic data regarding nAMD/DME treatment was available. Eligible patients were clustered into three groups: newly treated patients (defined as patients having received 3–6 injections and continuing IVT treatment), experienced patients (having received more than 6 injections and continuing therapy), and non-persistent patients (having discontinued their IVT therapy, defined as not having received any consultation by the study site for more than 6 months). After group assignment, SIs were asked to consecutively invite patients to participate in the study at the nearest opportunity, i.e., the next regular visit of a patient to the study site. Patients assigned to the non-persistent group were invited via postal mail. Only patients who gave their written informed consent were included in the study. Main sociodemographic and disease-related patient data were documented by participating study sites at the date of participant inclusion based on a pre-defined case report form using a web-based documentation tool. ABQ-IVT data were collected by means of structured phone interviews conducted by trained interviewers using a computer-assisted data collection tool. Patients were asked to answer additional questions regarding their quality of life, perceived eyesight, and dependency on support. Additionally, they were asked to assess their own treatment adherence and persistence.

The study was approved by the independent Ethics Commissions of the Universität Rostock, Germany (Registration Number: A 2018-0063).

### Evaluation of identified barriers

Outcomes of the ABQ-IVT were descriptively analyzed. The number and proportion of patients affected by each barrier as well as the number and proportion of patients affected by a minimum number of barriers were reported. For this, answers were dichotomized into "affected" and "not affected" where patients were considered to experience a barrier if their answer to the respective item was > 2 ("slightly disagree" or "strongly disagree" after reverse coding). The total score was calculated by adding the scores of each item. As it was assumed that the aspects investigated thus far are of similar importance, scores of each item were weighed equally.

### Cluster analysis

The dichotomized ABQ-IVT answers were used as predictors in a hierarchical cluster analysis using the Hamann dissimilarity measure for binary data and weighted-average linkage. The Duda–Hart Je(2)/Je(1) index and the associated pseudo-*T*-squared value were computed to determine the number of clusters. The identified clusters were described in terms of size, sociodemographic, DME/nAMD-related, and treatment-related characteristics as well as answers to interview questions. Differences between each cluster and all other patients were analyzed using Pearson's chi-squared test for binary variables and the non-parametric Mann–Whitney U-test for continuous variables.

The statistical analyses were conducted using STATA (StataCorp. 2015. Stata Statistical Software: Release 14. College Station, TX: StataCorp LP).

## Results

### Questionnaire development

The initially drafted ABQ-IVT contained 24 items that were subsequently evaluated in qualitative interviews with 15 patients. This draft appeared to contain only relevant and clearly formulated items from a patient's point of view; thus, no modifications were applied. The questionnaire applied in the patient interviews is provided in the supplementary material (Additional file 1: Table S1 [English] and Additional file 2: Table S2 [German]).

### Patient characteristics

A total of 253 patients were included in the study after recruitment in 13 different study sites. Nineteen patients did not complete the ABQ-IVT. No significant differences in terms of patient characteristics could be identified between patients having completed the questionnaire and those who refrained from completing it. The validation cohort consisted of 234 patients, including 78 DME patients and 156 nAMD patients. Patients with DME had a mean age of 63.5 years and 38.5% were female, while patients with nAMD were older (mean age: 78.2) and had a higher proportion of females (60.9%). The mean time since first diagnosis was approximately 3 years for both indications. The proportion of newly treated (32.1%–34.0%), experienced (63.4%–64.1%), and non-persistent patients (2.6%–3.8%) was similar in both groups. The mean number of IVTs per eye was higher in nAMD patients (12.2 injections) compared to DME patients (8.9 injections). Patient characteristics are detailed in Table 1.

**Table 1:** Patient characteristics

	DME	nAMD
N	78	156
Age in years at inclusion | mean (SD)	63.5 (11.7)	78.2 (8.3)
Female gender | n (%)	30 (38.5)	95 (60.9)
Newly treated patients | n (%)	25 (32.1)	53 (34.0)
Experienced patients | n (%)	50 (64.1)	99 (63.4)
Non-persistent patients | n (%)	3 (3.8)	4 (2.6)
Time since first diagnosis of DME/nAMD in years | mean (SD)	3.0 (3.1)	2.9 (3.1)
Both eyes affected | n (%)	49 (62.8)	52 (33.3)
VA LogMAR at start of treatment—treated eye | mean (SD)^a^	0.5 (0.2)	0.4 (0.2)
VA LogMAR at start of treatment—untreated eye | mean (SD)^b^	0.5 (0.4)	0.5 (0.3)
Duration of treatment in years | mean (SD)	1.9 (0.8)	1.9 (0.9)
Duration of treatment ≤ 12 months | n (%)	14 (17.9)	32 (20.5)
Number of injections per treated eye since start of therapy | mean (SD)	8.9 (5.5)^c^	12.2 (9.0)^d^
Catarakt | n (%)	40 (51.3)	53 (34.0)
Glaucoma | n (%)	8 (10.3)	19 (12.2)
Diabetic Retinopathy | n (%)	63 (80.8)	0 (0.0)
Patient reading capabilities assessed by physicians^e^		
Without magnifying glass | n (%)	39 (50%)	110 (70.5%)
With magnifying glass | n (%)	4 (5.1%)	16 (10.3%)
Not able to read | n (%)	0 (0%)	7 (4.5%)
Unknown | n (%)	35 (44.9%)	23 (14.7%)
Patient reading capabilities assessed by patients^c^		
Without magnifying glass | n (%)	59 (75.6%)	104 (66.7%)
With magnifying glass | n (%)	12 (15.4%)	40 (25.6%)
Not able to read | n (%)	7 (9%)	12 (7.7%)
Unknown | n (%)	0 (0%)	0 (0%)

^a^n = 325 eyes; ^b^n = 141 eyes; ^c^n = 125 eyes; ^d^n = 202 eyes; ^e^At the time of study inclusion/phone interview; SD, standard deviation; DME, diabetic macular edema; nAMD, neovascular age-related macular degeneration; VA, visual acuity; IVT, intravitreal anti-VEGF therapy

### Psychometric properties of the ABQ-IVT

After reversing the reverse-coded questionnaire items for homogenous item formulation, the distribution of data recorded for all items was right-skewed with the exception of Item 9 (‘Hope for healing'), which showed a left-skewed distribution. No missing data was detected as all interview participants were able to answer the 24 ABQ-IVT items. Reliability testing revealed a Cronbach's α of 0.73 and an average inter-item correlation of 0.10 for the original version, including 24 items.

To test whether to construct two distinct questionnaires for patients with nAMD and patients with DME, separate reliability analyses were run for each indication (Table 2). While inclusion of Item 4 (‘shared decision making') reduced questionnaire reliability when answered by the overall sample, it seemed to be a relevant item for the DME sample. Several items (7, ‘belief in need for therapy', 10, ‘unsatisfaction', 14, ‘cost of treatment') were less relevant for DME patients. After an in-depth discussion of the results, the expert panel decided to recommend the use of one "uniform" ABQ-IVT for both indications and to include Item 4. Removal of items with specifically low item-total correlations (Item 2, 5, 7, 8, 9, 10, 11, 13, and 16) lead to an increase in reliability of the questionnaire, measured by assessing Cronbach's α. Accordingly, the questionnaire was reduced by eliminating these questions, resulting in a 17-item ABQ-IVT with a Cronbach's α of 0.78. Item-total correlations of the reduced 17-item questionnaire ranged from 0.26 (item 4, ‘shared decision making') to 0.60 (item 22, ‘comorbidity'). Table 2 depicts the reliability analysis outcomes, including the results for each indication separately as well as the "overlap". The final 17-item ABQ-IVT (translated to English) can be found in Table 3 (for the original German version, refer to Additional file 3: Table S3).

**Table 2:** Reliability analysis of items included in the preliminary 24-item ABQ-IVT

		DME	nAMD	“Overlap"
	n	78	156	234
	Cronbach's α coefficient	0.81	0.79	0.78
Item	Abbreviation			
1	‘Information’	+	+	+
2	‘Education’	–	–	–
3	‘Trust in physician’	+	+	+
4	‘Shared decision making’	+	–	+
5	‘Need for compliance to appointments’	–	–	–
6	‘Discomfort in doctor's office’	+	+	+
7	‘Belief in need of therapy’	+	–	+
8	‘Positive treatment experience at start of therapy’	–	–	–
9	‘Hope for healing’	–	–	–
10	‘Unsatisfaction’	+	–	+
11	‘Immediate medical consultation in case of deterioration’	–	–	–
12	‘Depression’	+	+	+
13	‘Forgetfulness’	–	–	–
14	‘Cost of treatment’	–	+	+
15	‘Side effects’	+	+	+
16	‘Discussion of concerns with physician’	–	–	–
17	‘Time commitment’	+	+	+
18	‘Travel/opportunity costs’	+	+	+
19	‘Challenge accompanying person’	+	+	+
20	‘Burden for family members’	+	+	+
21	‘Lack of support’	+	+	+
22	‘Comorbidity’	+	+	+
23	‘Private/professional obligations’	+	+	+
24	‘Too old for therapy to be worthwhile’	+	+	+
	Number of items	16	14	17

"−"item excluded as exclusion led to an increase of Cronbach's alpha, " + " item kept

**Table 3:** Final 17-item Adherence Barriers Questionnaire for IVT (translated to English)

N	Item phrasing	Abbreviation
1	"I generally feel well informed about the treatment of my eye disease"	‘Information’
3	"I trust my eye doctor(s)"	‘Trust in physician’
4	"My eye doctor includes me in decisions about the course of treatment"	‘Shared decision making’
6	"I often feel uncomfortable in the doctor's office	‘Discomfort in doctor's office’
7	"Sometimes I am unsure whether the eye injections are indeed necessary"	‘Belief in need of therapy’
10	"I am dissatisfied with my current care/treatment"	‘Unsatisfaction’
12	"Generally, I often feel downcast and sometimes discouraged and depressed"	‘Depression’
14	"My injection treatments are tied to substantial costs for me"	‘Cost of treatment’
15	"I am afraid of the IV treatments and/or the side effects"	‘Side effects’
17	"Attending eye doctor appointments poses a high time burden (journey/waiting times) for me and/or my relatives"	‘Time commitment’
18	"Attending eye doctor appointments poses a high financial burden (e.g. travel costs/absenteeism) for me and/or my relatives"	‘Travel/opportunity costs’
19	"Especially doctor's appointments which require an accompanying person pose a challenge"	‘Challenge accompanying person’
20	"I am worried about being a burden to my family/relatives and to have to ask for help"	‘Burden for family members’
21	"I would need help on a daily basis (particularly in context of healthcare). However, I do not receive any"	‘Lack of support’
22	"Apart from my eye condition I experience other conditions which hampers my attendance to appointments"	‘Comorbidity’
23	"I have private/professional duties which are hardly compatible with the treatment of my eye disease"	‘Private/professional obligations’
24	"Due to my old age, I am unsure whether the efforts associated with my IV treatment are worth it"	‘Too old for therapy to be worthwhile’

In an attempt to externally validate the ABQ-IVT, we assessed the correlation of scores with patient interview questions assessing treatment adherence. When asked how they rated their own medication adherence on a scale from 0 to 100, 191 patients (82%) assessed themselves at exactly 100. These patients had a mean ABQ-IVT score of 21.5, while the remaining patients who rated their adherence to being less than 100 had a mean score of 26.2. This difference was statistically significant (p = 0.017). No significant score difference was found between patients who never missed a doctor's appointment and patients who missed an appointment in the past.

Based on the reduced scale with 17 items, factor analysis showed no evidence for subscales of the ABQ-IVT, as only one factor was identified in the analysis.

### Identified barriers

**Fig. 1:**
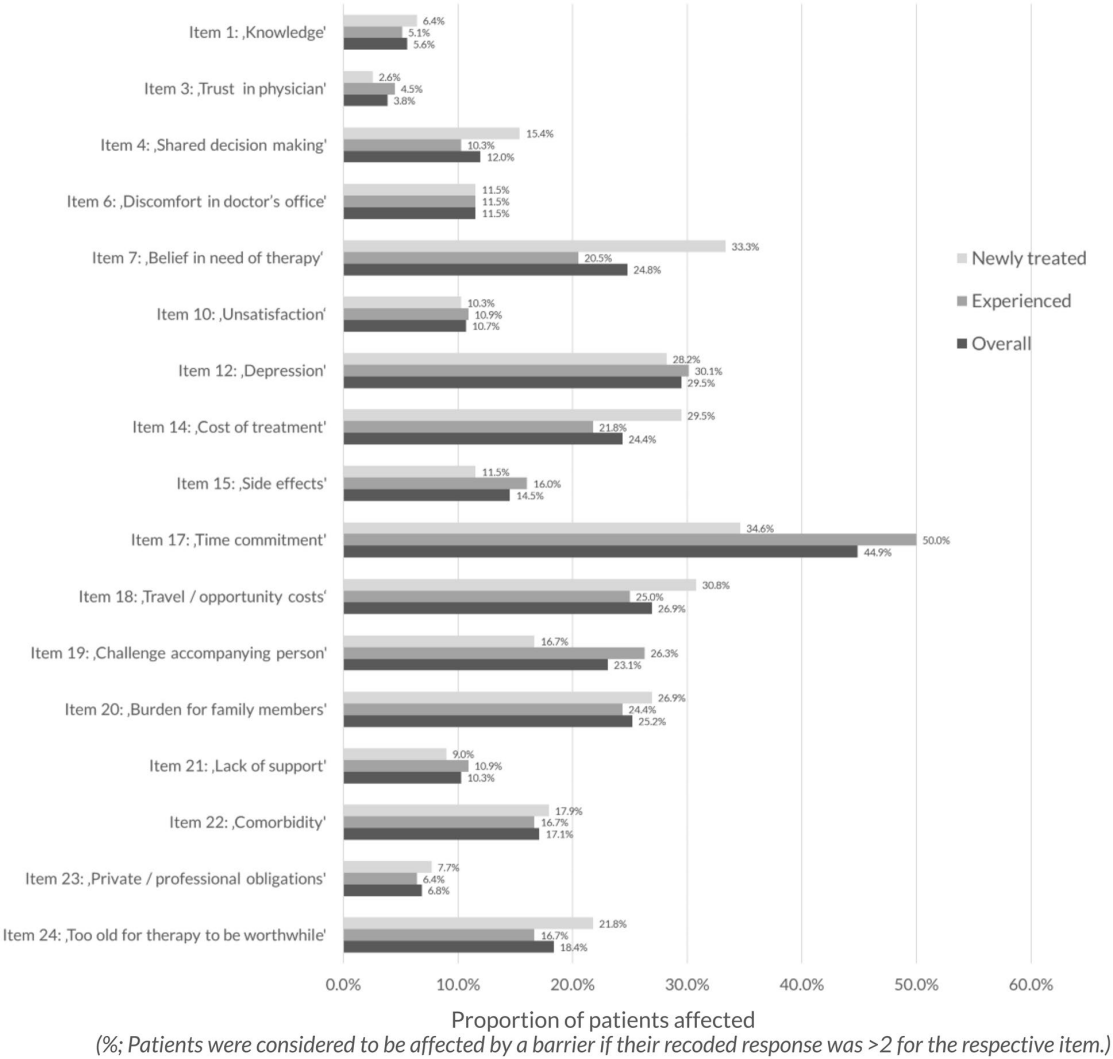
Proportions of patients affected by each barrier of the ABQ-IVT

By far, the most prominent barrier was Item 17 (‘time commitment'), with 105 patients (44.9%) stating that complying with ophthalmologist appointments is linked to a high time burden for themselves or their relatives (Fig. 1). The barrier is even more prominent in treatment-experienced patients compared to newly treated patients (50.0% versus 34.6% of patients affected by this barrier). Between 23.1% and 29.5% often felt discouraged or depressed, experienced a high burden due to travel and opportunity costs, were unsure whether IVT is necessary, were afraid of being a high burden to their relatives, experienced high costs regarding the treatment itself, and/or were especially challenged by appointments requiring an accompanying person. A lack of belief in need of the therapy as well as of shared decision making is more prevalent in newly treated patients than in treatment-experienced patients (33.3% versus 20.5% and 15.4% versus 10.3%). Comorbidities, fear of injections or side effects, and doubt about the worth of therapy due to old age were barriers for 15–18% of participants. Lack of information about the disease, lack of clarification by the treating physician, and private/occupational duties represented a barrier for a few patients (4–7%).

**Fig. 2:**
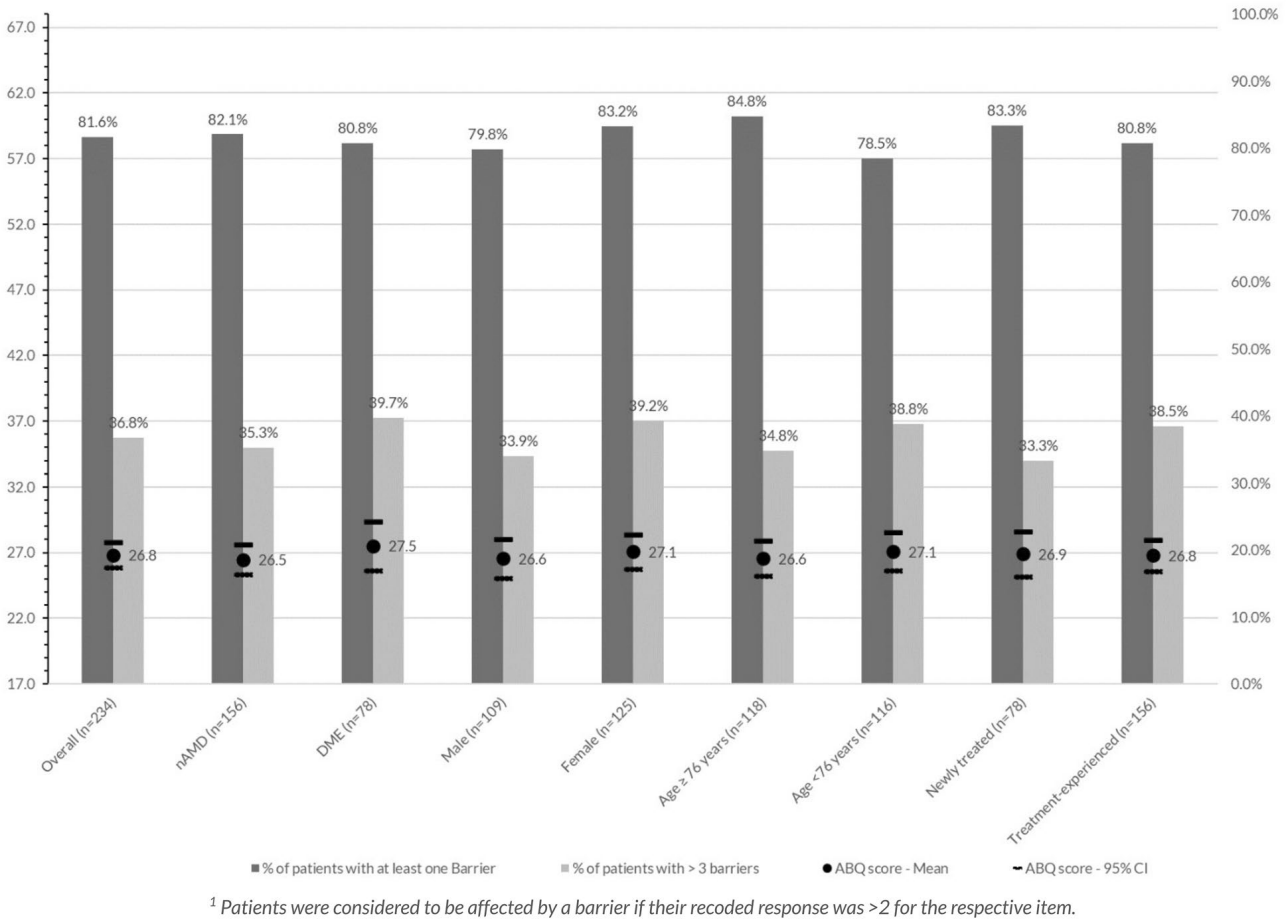
Proportions of patients affected by a number of barriers

Almost one-fifth of the participants did not experience any of the barriers addressed in the ABQ-IVT, while 82% were affected by at least one barrier (Fig. 2). Approximately one-third of the patients indicated they experienced more than three barriers. The average ABQ-IVT score in the overall sample was 26.8 (95%-CI: 25.8–27.8), with showing a higher mean in DME patients compared to nAMD patients (27.5 versus 26.5).

### Cluster analysis

**Fig. 3:**
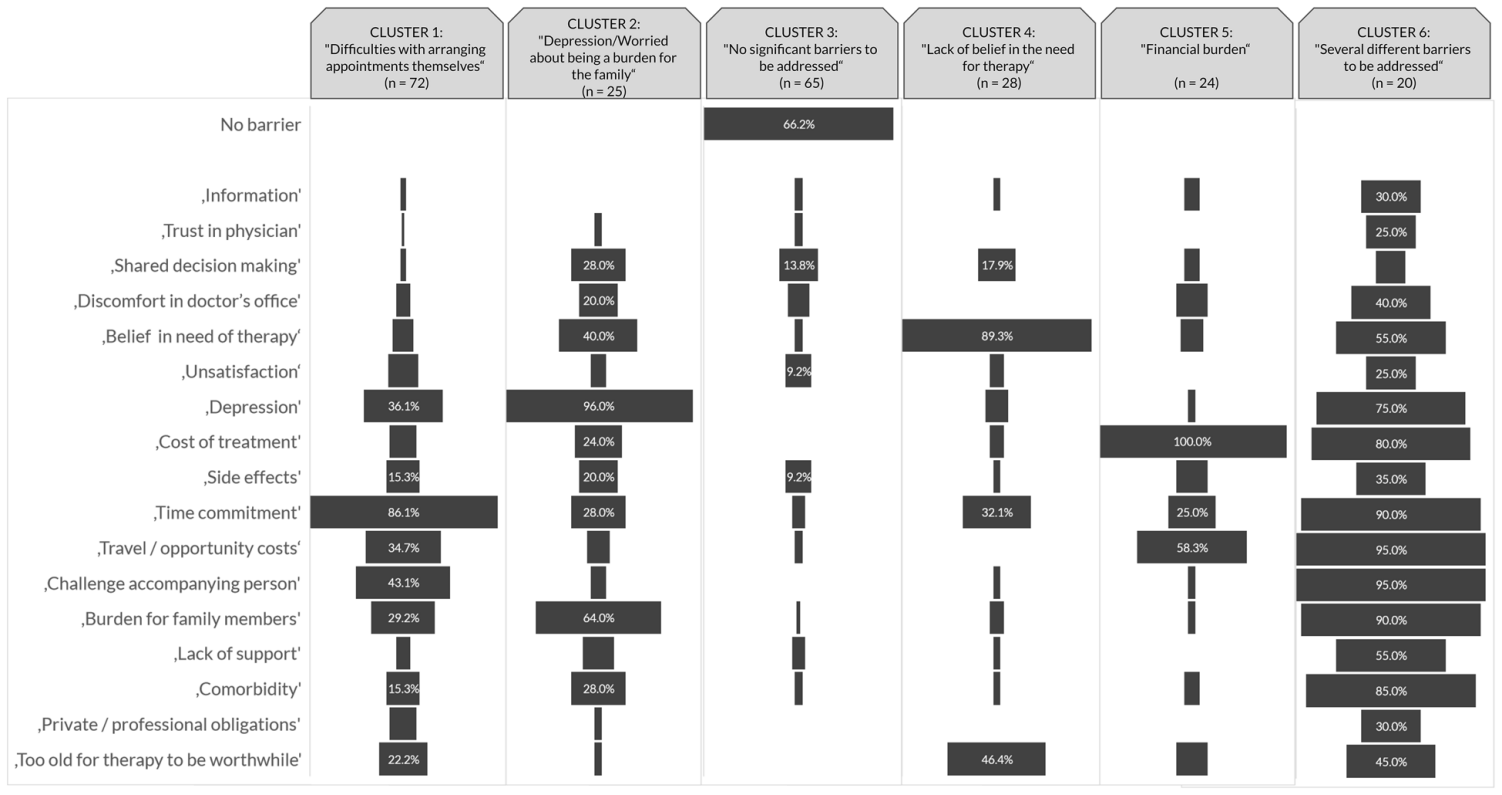
Percentage of patients who experienced each barrier within a given cluster

Six patient clusters were identified in a cluster analysis using the ABQ-IVT responses as parameters for patient assignment. Cluster size ranged from 20 patients (8.5%, cluster 6) to 72 patients (30.8%, cluster 1), with clusters 1 and 3 being composed of 65 or more patients and clusters 2, 4, 5, and 6 representing less than 30 patients. Proportions of patients experiencing a certain barrier within a given cluster are shown in Fig. 3. At least a quarter of patients experienced high time burden in all clusters, with cluster 3 being the only exception.

Patients of cluster 1 seemed to mainly have struggled with arranging the appointment itself: Many experienced a high time burden (86.1%), and almost half of this group was particularly challenged by appointments requiring an accompanying person (43.1%). Another difference compared to other clusters was that approximately a third of the patients in cluster 1 generally felt downcast (36.1%), experienced high travel/opportunity costs (34.7%) and/or were worried about being a burden to family members (29.2%).

Cluster 2 was mainly characterized by patients feeling downcast (96.0%) and two-thirds of this group worried about being a burden to family members (64.0%). More than a third of this cluster doubted the necessity of IVT (40.0%).

Patients in cluster 3 experienced few barriers: Five out of 17 barriers were not experienced by any one of this group and only one item (‘shared decision making', 13.8%) was a barrier for more than 10%. Cluster 3 was the only segment in which few patients (4.6%) struggled with the time commitment.

Most members of cluster 4 doubted the necessity of IVT (89.3%) and almost half of this group believed they are too old for the therapy to be worthwhile (46.4%).

Patients of cluster 5 mainly struggled with costs: All patients of this group (100.0%) experienced high costs of the treatment itself, while more than half (58.3%) additionally experienced high travel/opportunity cost.

Cluster 6 was characterized by patients experiencing a variety of barriers: More than half of the items (nine) presented a barrier for more than half the group and no barrier was experienced by less than 15%. Most prominent barriers were ‘travel/opportunity costs’ (95%), ‘challenge accompanying person’ (95%), ‘burden for family members’ (90%), and ‘time commitment’ (90%). Cluster 6 was the only segment in which comorbidities (85%) and lack of support (55%) represent considerable barriers. Cluster 6 is the smallest of the six groups (n = 20).

Table 4 shows the identified patient clusters in terms of sociodemographic, indication-related, and treatment-related characteristics as well as answers to interview questions. Patients of cluster 1 who mainly experienced difficulties with arranging the appointments themselves reached a significantly higher mean ABQ score (24.2 vs. 21.5, p < 0.001) and received more injections per study eye on average (13.8 vs. 9.8, p = 0.001).

**Table 4:** Description of identified patient clusters in terms of patient characteristics and answers to interview questions

Cluster (size)	Cluster 1 (n = 72, 30.7%)	Cluster 2 (n = 25, 10.7%)	Cluster 3 (n = 65, 27.8%)	Cluster 4 (n = 28, 12.0%)	Cluster 5 (n = 24, 10.3%)	Cluster 6 (n = 20, 8.5%)	Overall (n = 234)
Short description of identified clusters	“Difficulties with arranging the appointments themselves”	“Depression/Worried about being a burden for the family”	“No significant barriers to be addressed”	“Lack of belief in the need for therapy”	“Financial burden”	"Several different barriers to be addressed “	
Mean (SD)							
ABQ score	24.21 (SD: 4.65)***	24.08 (SD: 5.84)*	16.46 (SD: 2.95)***	19.00 (SD: 2.96)*	22.33 (SD: 4.34)	37.05 (SD: 4.9)***	22.32 (SD: 6.95)
Age [years]	71.13 (SD: 13.26)	72.4 (SD: 13.73)	72.4 (SD: 11.09)	76.39 (SD: 9.87)	78.67 (SD: 8.84)**	74.35 (SD: 9.36)	73.29 (SD: 11.79)
Time since diagnosis [years]	3.08 (SD: 2.70)	3.64 (SD: 4.46)	2.69 (SD: 2.90)	1.97 (SD: 1.10)	3.46 (SD: 4.36)	3.13 (SD: 3.02)	2.94 (SD: 3.10)
Time since first IV [years]	1.63 (SD: 1.12)	1.42 (SD: 1.00)	1.74 (SD: 0.98)	1.74 (SD: 1.06)	1.73 (SD: 0.98)	1.37 (SD: 0.91)	1.64 (SD: 1.03)
Number of injections per treated eye	13.83 (SD: 9.13)**	10.14 (SD: 6.97)	9.62 (SD: 6.25)	8.43 (SD: 5.89)*	11.42 (SD: 6.94)	10.13 (SD: 6.88)	11.06 (SD: 7.6)
Feeling at time of interview^a^	3.17 (SD: 0.69)	3.4 (SD: 0.91)*	2.83 (SD: 0.78)**	2.79 (SD: 0.83)*	3.29 (SD: 0.69)	3.35 (SD: 0.67)	3.08 (SD: 0.78)
Change in VA^b^	0.04 (SD: 0.22)	0.00 (SD: 0.24)	0.06 (SD: 0.18)	0.03 (SD: 0.18)	− 0.01 (SD: 0.23)	0.03 (SD: 0.22)	0.04 (SD: 0.21)
Perceived change in eyesight^c^	0.90 (SD: 22.27)	-2.37 (SD: 21.43)	5.33 (SD: 23.18)**	− 2.20 (SD: 10.76)	8.05 (SD: 23.98)	− 12.88 (SD: 20.36)**	0.94 (SD: 21.97)
n (%)							
nAMD (the other cases are DME)	41 (56.9%)*	20 (80.0%)	41 (63.1%)	21 (75.0%)	20 (83.3%)	13 (65.0%)	156 (66.7%)
Female	37 (51.4%)	16 (64.0%)	32 (49.2%)	16 (57.1%)	15 (62.5%)	9 (45.0%)	125 (53.4%)
Both eyes affected	31 (43.1%)	13 (52.0%)	30 (46.2%)	7 (25.0%)*	11 (45.8%)	9 (45.0%)	101 (43.2%)
Rate own adherence as 100%	63 (87.5%)	19 (76.0%)	52 (81.3%)	27 (96.4%)*	19 (79.2%)	11 (55.0%)**	191 (82.0%)
Quality of life affected strongly^d^	26 (36.6%)	17 (70.8%)***	11 (17.5%)**	6 (21.4%)	5 (20.8%)	14 (73.7%)***	79 (34.5%)
Need or prefer daily help^e^	23 (32.4%)	13 (52.0%)*	14 (22.6%)*	6 (23.1%)	6 (28.6%)	12 (60.0%)**	74 (32.9%)
Cataract	31 (43.1%)	8 (32.0%)	24 (36.9%)	8 (28.6%)	12 (50.0%)	10 (50.0%)	93 (39.7%)
Glaucoma	8 (11.1%)	6 (24.0%)*	6 (9.2%)	4 (14.3%)	1 (4.2%)	2 (10.0%)	27 (11.5%)
Diabetic retinopathy	26 (36.1%)*	4 (16.0%)	19 (29.2%)	4 (14.3%)	4 (16.7%)	6 (30.0%)	63 (26.9%)
Cancer	4 (5.6%)	1 (4.0%)	0 (0.0%)*	2 (7.1%)	2 (8.3%)	2 (10.0%)	11 (4.7%)

Percentages of categorical variables may not correspond to the fraction of the cluster size as a number of patients refused to answer / did not know the answer to some of the interview questions. Significant α = 0.05 for comparisons of the respective cluster with all other patients; * *p* value < 0.05, ** *p* value < 0.01, *** *p* value < 0.001; significant comparisons made bold. ^a^Patients were asked to rate how they currently felt on a scale from 1 = Excellent to 5 = Bad. ^b^Change from first intravitreal injection to study interview. ^c^Patients were asked to rate their eyesight at therapy start and at the time of interview on a scale from 0 to 100. ^d^Patients were asked to what extent their disease limits their quality of life; answers were 1 = Not at all, 2 = Rather not, 3 = Somehow, and 4 = Strongly. For analysis, the variable was dichotomized into strongly affected (4) and not strongly affected patients (1, 2, 3). ^e^ Patients were asked whether they needed help in daily life; answers were 1 = Yes, in almost any situation, 2 = Yes, in some particular situations, 3 = I manage on my own but prefer assistance, 4 = No support needed. For analysis, this variable was dichotomized into a group needing no support (4) and a group needing or preferring support (1, 2, 3). DME, diabetic macular edema; nAMD, neovascular age-related macular degeneration; SD, standard deviation; VA, visual acuity

The despondence of patients in cluster 2 is reflected by their interview responses: When asked how they felt between 1 = Excellent and 5 = Bad, the average response was 3.4 (vs. 3.0, p = 0.021), and 70.8% of patients (vs. 30.2%, p < 0.001) stated that their disease strongly affects their quality of life. Half of this patient segment needed or preferred daily help (52.0% vs. 30.5%, p = 0.031).

Patients of cluster 3 generally experienced few barriers, which is reflected by their low mean ABQ score (16.5 vs. 24.6, p < 0.001). They felt better at the time of the interview compared to the rest of the sample (2.8 vs. 3.2, p = 0.002), and few stated that their quality of life was affected strongly by their condition (17.5% vs. 41.0%, p = 0.001). On average, they also reported a more positive change in perceived eyesight from the treatment start to the time of the interview compared to other patients (+ 5.3 vs. – 0.7, p = 0.003), even though their measured visual acuity difference did not differ significantly from other groups.

Patients doubting the therapy necessity (cluster 4) received significantly fewer injections on average compared to other patients (8.4 vs. 11.4, p = 0.049). However, no difference was detected for the proportion of patients having missed an appointment or having visited the office more than a month ago (data not shown). A smaller proportion of cluster 4 had both eyes affected by their condition compared to other groups (25% vs. 45.6%, p = 0.039) and a higher proportion rates their own adherence to be 100% (96.4% vs. 80.0%, p = 0.034).

Patients experiencing a high cost burden (cluster 5) were significantly older on average (78.7 vs. 72.7, p = 0.007).

Finally, the prominence of barriers in cluster 6 is reflected by their high mean ABQ score (37.1 vs. 20.9, p < 0.001) and their low self-reported adherence (55% reporting 100% adherence vs. 84.5%, p = 0.001). Many patients of this segment stated that their quality of life is strongly affected by the condition (73.7% vs. 31.0%, p < 0.001) and they felt worse on average (3.4 vs. 3.1, p = 0.074), even though this finding did not cross the significance threshold. Mean perceived eyesight decreased in cluster 6 significantly more than in other patients (–12.9 vs. + 2.3, p = 0.002). This segment also contained more patients needing or preferring daily help (60.0% vs. 30.2, p = 0.007).

## Discussion

This validation study confirmed previous evidence that a large number of relevant barriers to IVT are encountered by respective patients. Scope and extent can explain non-adherence and non-persistence to a high degree. Once the ABQ-IVT questionnaire has been developed and validated, a valuable and effective tool will be available that can be used immediately in everyday life for DME and nAMD patients. The previously established ABQ was successfully adapted to reflect the challenges faced by these patients. By far, the most frequently reported barrier was time commitment, which challenged almost half of the participating patients. Additionally, we identified six different patient clusters, which can give valuable insights into potential existing patient subpopulations. Our findings also indicate that many treating ophthalmologists are already working successfully in helping their patients understand their care.

### Psychometric properties

To our knowledge, the ABQ-IVT is the first questionnaire addressing the nature of barriers preventing DME and/or nAMD patients from adhering to their recommended IVT. No previous studies have investigated PROs focusing on adherence barriers in these patients at the time of study start. The final 17-item ABQ-IVT showed reasonable internal reliability and may be used to assess the adherence of both DME and nAMD patients. No subscales were identified. Previous versions of the ABQ could be divided into subscales of intentional, unintentional, and medication/healthcare system-related barriers [25, 26]. However, anti-VEGF therapy with regular injections poses specific challenges to DME/nAMD patients in terms of travel and interference with daily life (e.g., eyesight difficulties after injection) [30]. Therefore, many additional items were needed to adequately assess the adherence of these patients. After reliability analysis, the ABQ-IVT contained only seven items that were derived from the original ABQ while ten treatment-specific items were added. The rigorous change in questions most likely hindered the identification of analogous subscales.

### Identified barriers

The special challenges faced by patients requiring IVT were additionally reflected by the questionnaire responses collected from our participants: The most frequently reported barrier was time commitment, followed by low moods. Approximately a quarter of patients were particularly challenged by appointments requiring an accompanying person and/or felt they were a burden to family members, illustrating the dependency of patients requiring IVT on support from relatives and friends and the resulting psychological burden posed by this dependency [30]. Costs for the treatment itself, as well as travel/opportunity costs, were also experienced by a quarter of patients. Another 25% doubted the necessity of IVT. Many of these most prominent barriers are indication-specific, as they reflect the challenges posed by the required travel to the study site. Other studies have identified similar barriers to IVT adherence, such as time and financial burden, comorbidities, and disbelief in therapy [22, 31]. Another study identified reasons for discontinuation of IVT (non-persistence), including fear of injection in addition to disbelief in the therapeutic benefits and financial limitations [32].

### Cluster analysis

We identified six potential patient segments within our study. Patients receiving a high number of injections were particularly challenged by the arrangement of doctor's appointments, e.g., the required time, company, and costs. Another patient segment was characterized by low moods and the feeling of being a burden to family members. A higher proportion of this group needed or preferred daily support and felt that their condition strongly affects their quality of life. The same held true for another small patient segment, which was characterized by patients experiencing a high number of adherence barriers; these patients additionally indicated drastic eyesight worsening since therapy start, suggesting a pessimistic view of these patients on the progression of their condition. Considering that the quality of life is specifically affected in these patients, special attention needs to be paid to the psychological health of DME and nAMD patient subpopulations, especially those who require daily support. Almost a third of the investigated patient sample experienced very few barriers, feeling better overall, and indicating little impact on their quality of life. Patients of this group may generally have a more positive outlook regarding their disease as they perceived a positive change in their eyesight since therapy started. Furthermore, older patients in particular may experience high costs associated with travel/opportunity costs as well as the treatment itself. Our analysis illustrates the variety of patient profiles; physicians are faced with the challenge of identifying patients' constitution and taking appropriate action if needed.

## Limitations

Several limitations need to be acknowledged. The main limitation of this study is the absence of a standardized adherence measure for external consistency validation. Initially, we planned to assess adherence with the recommended treatment regimen (as specified by the treating physician) based on visits of a patient to the respective study site. Treatment schemes differed between study centers (e.g., appointment arrangement based on the progression of the disease with different time intervals) and even within one study center. With few patients following a fixed treatment regime, no patient-individual non-adherence could be defined. Even though we found an indication for a relationship between ABQ-IVT scores and self-reported adherence, a comparison with a validated adherence measure is missing. Given the difficulties in capturing non-adherence as an external criterion, this evaluation has been limited to the internal validity of the instrument. Secondly, the cross-sectional design did not allow for the assessment of test–retest reliability (ability to detect changes). Thirdly, even though the ABQ-IVT was based on a well-established instrument for adherence barrier measurement and potential causes of non-adherence in nAMD/DME patients were discussed within an expert panel, the possibility exists that our instrument does not cover all existing adherence barriers faced by these patients. Further research is needed to fully prove the content validity of the ABQ-IVT and to what extent the questionnaire explains existing non-adherence. Moreover, we sought to prevent bias arising from consecutive enrollment by identifying eligible patients based on existing medical data and contacting non-persisting patients via post mail. Nonetheless, a risk of selection bias remains since fewer patients of this group consented to the study, and consequently, patients with more regular visits were more likely to be overrepresented. Additionally, even if the study explicitly aimed to include patients who discontinued the therapy and who were invited separately via a written invitation letter, only seven non-persistent patients participated in our survey. With respect to potential reasons for the poor recruitment of non-persistent patients, consideration should be given to the possibility of better methodology for future studies. For example, it could be considered whether a different access route (recruitment independent of the treating physicians), alternative methods of informed consent (by telephone/orally), and special forms of approaching patients could increase involvement of treatment dropouts.

Despite the potential selection bias, we found the age and gender distribution to be generally similar to that described in the literature [33–39]. Finally, the groups generated via cluster analysis can only give a hint of potential existing patient subpopulations. Even though trends in terms of dichotomized ABQ-IVT answers could be observed within the clusters, no group is homogenous in terms of their answers, i.e., patients and their indicated barriers differ within one cluster.

### Practical implications

The questionnaire can be used immediately in daily practice. The active querying of possible hurdles can reveal hidden problems that patients and their relatives are not addressing by themselves. This gives those affected the reassurance that the focus is not limited to the eye and the retina, but that the practitioner is interested in the well-being of his or her patient beyond the medical aspects. In addition, it is likely that completion of the ABQ-IVT improves health literacy on the patient’s side, which in turn could have a positive effect on adherence [40].

Most importantly, there is a great opportunity if physicians align their reactions with the identified barriers. Therefore, it is crucial for caretakers to be aware of the challenges (including their severity and scope) faced by patients, to be able to address them in patient consultations, and to encourage and emphasize the importance of treatment continuation despite the challenges faced. Personalized information and needs-based interactions should improve the understanding of the permanent need for re-treatment, especially in view of the deficits that current sources of information still have [41]. Factors such as higher age, worse VA at treatment initiation, or higher distance to the treatment center, should be considered carefully to get an approximation of a patient’s risk for non-adherence.

Time commitment and associated opportunity costs depict challenges faced by a substantial proportion of patients receiving IVT. Even if frequent re-treatments are indispensable, optimizing the organizational measures of everyday treatment could lead to the reduction of required physician visits, if room for improvement exists at the respective site. The substantial amount of patients challenged by treatment costs suggests that a change in cost coverage is needed. Moreover, Depression depicts one of the barriers which can directly be addressed while being one of the strongest predictors of medication non-adherence [23, 42]. In an attempt to make non-adherence more addressable for treating doctors, Devine et al*.* [23] have rephrased common barriers to represent physician/health system hurdles. In this sense, physicians are urged to be watchful of patients who may experience or be susceptible to developing depression and apply suitable screening tools and/or provide an appropriate referral when needed. Furthermore, physicians have the opportunity to address patients' disbelief in therapy by informing doubtful patients about the treatment rationale and emphasizing the necessity and potential benefits of the intervention [43]. Generally, communication in medical care and positive physician–patient partnerships are correlated with higher patient adherence [44, 45]. In this study, only 6% of included patients have suggested problems with understanding their disease or treatment as a barrier to therapy adherence, indicating that physicians adequately educate their patients on their condition and related care. Nonetheless, although barriers such as misinformation, distrust in the physician, lack of shared decision making, fear of side effects, and discomfort in the doctor’s office have been reported less often, these depict substantial barriers for treatment adherence, if present. Thus, knowledge, detection, and addressing of these hurdles by the treating physician is crucial.

Should the hypothesis of certain patient typologies be confirmed by further data, patient characteristics could be used to identify these subgroups and thus to keep awareness of disruptive factors and negative attitudes hindering adherence within the respective groups. It is not due to a lack of opportunity to detect undertreatment, but rather to take action before unwanted delays, breaks, and discontinuations occur. High-quality knowledge about effective intervention (communication and organization) is still lacking, but this questionnaire offers opportunities to initiate prospective studies and to use validated items for this purpose.

## Conclusion

The original ABQ was successfully adapted for nAMD and DME patients requiring IVT. The developed ABQ-IVT is a reliable instrument that adequately reflects the special challenges faced by these patients. IVT injections must be administered at a doctor's office or care center, and the resulting travel requirements present several hurdles for affected patients. Patient subpopulations face different barriers and may, therefore, need distinct care. It is important for treating physicians to understand the diverse challenges faced by patients requiring IVT, to be able to address these either directly or through patient consultations. As a substantial proportion of patients shows signs of depression, physicians are urged to pay special attention to this patient subpopulation and take action when needed.

The ABQ-IVT provides a practicable tool that could be used in clinical routine to identify potential adherence barriers in good time and, thus, to achieve the opportunity of early implementation of preventive measures in order to reduce the risk of non-adherence and non-persistence.

## Supplementary Information

**Additional file 1: Table S1.** Validated 24-item Adherence Barriers Questionnaire for IVT (translated to English).

**Additional file 2: Table S2.** Validated 24-item Adherence Barriers Questionnaire for IVT (German).

**Additional file 3: Table S3.** Final 17-item Adherence Barriers Questionnaire for IVT (German).

## Data Availability

The datasets generated and/or analyzed during the current study are not publicly available due to data protection guidelines.

## Abbreviations

ABQ: Adherence Barriers Questionnaire
DME: Diabetic macular edema
IVT: Intravitreal anti-VEGF injection
NA: Non-adherence
nAMD: Neovascular age-related macular degeneration
PRO: Patient-reported outcome
SI: Study investigators
VA: Visual acuity
VEGF: Vascular endothelial growth factor
