# Supplementary material for: Questionnaire for the assessment of adherence barriers of intravitreal therapy: the ABQ-IVT
Source: Int J Retina Vitreous. 2021 Jun 2;7:43. doi: 10.1186/s40942-021-00311-x (PMC8170736; doi:10.1186/s40942-021-00311-x)
Supplement: Supplementary file 1 — Additional file 1: Table S1. Validated 24-item Adherence Barriers Questionnaire for IVT (translated to English). [file 40942_2021_311_MOESM1_ESM.docx]

Table S1: Validated 24-item Adherence Barriers Questionnaire for IVT (translated to English)

| **N°** | **Item phrasing** | **Abbreviation** |
| --- | --- | --- |
| 1 | "I generally feel well informed about the treatment of my eye disease." | ‚Information' |
| 2 | „I generally consult my doctor regarding the planned course of treatment.” | 'Education ' |
| 3 | "I trust my eye doctor(s)." | ‚Trust in physician' |
| 4 | "My eye doctor includes me in decisions about the course of treatment." | ‚Shared decision making ' |
| 5 | „For the treatment to be successful, it is important that I adhere to agreed appointments.” | ‚Need for compliance to appointments' |
| 6 | "I often feel uncomfortable in the doctor's office." | ‚Discomfort in doctor's office' |
| 7 | "Sometimes I am unsure whether the eye injections are indeed necessary." | ‚Belief in need of therapy ' |
| 8 | „I have noticed an improvement in eyesight at the start of injection treatment. | ‚Positive treatment experience at start of therapy’ |
| 9 | „I hope that my eye disease will be cured in the near future and that I will not need any additional eye injections.” | ‚Hope for healing ' |
| 10 | "I am dissatisfied with my current care/treatment." | ‚Unsatisfaction ' |
| 11 | „As soon as I notice a deterioration of my eyes, I am immediately consulting my doctor.” | ‚Immediate medical consultation in case of deterioration' |
| 12 | "Generally, I often feel downcast and sometimes discouraged and depressed." | ‚Depression' |
| 13 | „I often forget things during my daily routine.“ | ‚Forgetfulness' |
| 14 | "My injection treatments are tied to substantial costs for me." | ‚Cost of treatment' |
| 15 | "I am afraid of the IV treatments and/or the side effects." | ‚Side effects' |
| 16 | „I am openly discussing my concerns regarding the treatment of my eyes with my physician.“ | 'Discussion of concerns with physician' |
| 17 | "Attending eye doctor appointments poses a high time burden (journey/waiting times) for me and/or my relatives." | ‚Time commitment' |
| 18 | "Attending eye doctor appointments poses a high financial burden (e.g. travel costs/absenteeism) for me and/or my relatives." | ‚Travel / opportunity costs' |
| 19 | "Especially doctor's appointments which require an accompanying person pose a challenge." | ‚Challenge accompanying person' |
| 20 | "I am worried about being a burden to my family/relatives and to have to ask for help." | ‚Burden for family members' |
| 21 | "I would need help on a daily basis (particularly in context of healthcare). However, I do not receive any." | ‚Lack of support' |
| 22 | "Apart from my eye condition I experience other conditions which hampers my attendance to appointments." | ‚Comorbidity' |
| 23 | "I have private/professional duties which are hardly compatible with the treatment of my eye disease." | ‚Private / professional obligations' |
| 24 | "Due to my old age, I am unsure whether the efforts associated with my IV treatment are worth it." | ‚Too old for therapy to be worthwhile' |
