# Supplementary material for: Questionnaire for the assessment of adherence barriers of intravitreal therapy: the ABQ-IVT
Source: Int J Retina Vitreous. 2021 Jun 2;7:43. doi: 10.1186/s40942-021-00311-x (PMC8170736; doi:10.1186/s40942-021-00311-x)
Supplement: Supplementary file 3 — Additional file 3: Table S3. Final 17-item Adherence Barriers Questionnaire for IVT (German). [file 40942_2021_311_MOESM3_ESM.docx]

Table S3: Final 17-item Adherence Barriers Questionnaire for IVT (German)

| **N°** | **Item phrasing** | **Abbreviation** |
| --- | --- | --- |
| 1 | „Grundsätzlich fühle ich mich über die Behandlung meiner Augenerkrankung sehr gut informiert.“ | ‚Information' |
| 3 | „Ich vertraue meinem/meinen Augenarzt/Augenärzten.” | ‚Trust in physician' |
| 4 | „Mein Augenarzt entscheidet gemeinsam mit mir über den weiteren Behandlungsverlauf.” | ‚Shared decision making ' |
| 6 | „In der Augenarztpraxis fühle ich mich oft unbehaglich.” | ‚Discomfort in doctor's office' |
| 7 | „Ich bin manchmal unsicher, ob die Spritzen ins Auge tatsächlich erforderlich sind.” | ‚Belief in need of therapy ' |
| 10 | „Ich bin mit meiner aktuellen Betreuung/ Behandlung unzufrieden.“ | ‚Unsatisfaction ' |
| 12 | „Ganz allgemein fühle ich mich doch oft niedergeschlagen, manchmal auch entmutigt und deprimiert.“ | ‚Depression' |
| 14 | „Mit meiner Spritzenbehandlung sind für mich erhebliche Kosten verbunden.” | ‚Cost of treatment' |
| 15 | „Ich habe große Angst vor der Spritzenbehandlung bzw. deren Nebenwirkungen.” | ‚Side effects' |
| 17 | „Die Wahrnehmung meiner Augenarzttermine ist für mich/meine Angehörigen mit einer hohen zeitlichen Belastung (Anfahrt-/ Wartezeiten) verbunden.“ | ‚Time commitment' |
| 18 | „Die Wahrnehmung meiner Augenarzttermine ist für mich/meine Angehörigen mit einer hohen finanziellen Belastung (z.B. Fahrkosten, Arbeitsausfall) verbunden.” | ‚Travel / opportunity costs' |
| 19 | „Insbesondere stellen Arzttermine, bei welchen ich eine Begleitperson benötige, eine große Herausforderung dar.” | ‚Challenge accompanying person' |
| 20 | „Ich habe Sorge, meiner Familie/meinen Angehörigen zur Last zu fallen und um Hilfe bitten zu müssen.” | ‚Burden for family members' |
| 21 | „Im Alltag (und speziell im Zusammenhang mit meiner gesundheitlichen Versorgung) bräuchte ich Unterstützung. Diese bekomme ich jedoch leider nicht.” | ‚Lack of support' |
| 22 | „Neben meiner Augenerkrankung habe ich weitere Erkrankungen, die es erschweren, regelmäßige Augenarzttermine wahrzunehmen.” | ‚Comorbidity' |
| 23 | „Ich habe private/berufliche Verpflichtungen, die schwer mit der Behandlung meiner Augenerkrankung vereinbar sind.” | ‚Private / professional obligations' |
| 24 | „Aufgrund meines Alters bin ich unsicher, ob sich der Aufwand lohnt, der mit der Spritzenbehandlung verbunden ist.” | ‚Too old for therapy to be worthwhile' |
